# Change in Lung Fluid Volume during Exercise in Patients with Exercise-Induced Mitral Regurgitation

**DOI:** 10.3390/medicina58060724

**Published:** 2022-05-28

**Authors:** Teruhiko Imamura, Masakazu Hori, Shuhei Tanaka, Nikhil Narang, Koichiro Kinugawa

**Affiliations:** 1 Second Department of Internal Medicine, University of Toyama, Toyama 930-0194, Japan; masahori6059@yahoo.co.jp (M.H.); stanaka@med.u-toyama.ac.jp (S.T.); kinugawa-tky@umin.ac.jp (K.K.); 2 Advocate Christ Medical Center, Oak Lawn, IL 60453, USA; nikhil.narang@gmail.com

**Keywords:** valvular disease, heart failure, congestion, hemodynamics

## Abstract

Exercise-induced mitral regurgitation (MR) can be diagnosed during stress echocardiography testing. Remote dielectric sensing (ReDS^TM^) is a noninvasive electromagnetic-based modality to measure lung fluid levels. The change in lung fluid levels in patients with MR during stress echocardiography remains unknown. Patients with symptomatic MR at baseline and suspected worsening exercise-induced MR underwent stress echocardiography. ReDS values were measured before and after the tests. A total of four patients (ages ranging between 74 and 84 years old, three women) underwent stress echocardiography testing using a bicycle ergometer. In patient A, MR effective regurgitant orifice area (EROA) remained unchanged and ReDS values decreased. EROA increased significantly with a small incremental change in ReDS values in patient B and patient C, who underwent valve repair with MitraClip later. Patient D had a mild increase in MR EROA but a considerable increase in ReDS values (from 22% to 32%), and eventually received valve repair with MitraClip. The ReDS system may be a complementary tool to conventional stress echocardiography in the evaluation of clinically significant MR and considering mitral valve intervention.

## 1. Introduction

Some patients may have severe dyspnea upon exertion due to mitral regurgitation (MR), which worsens during exercise [1]. This pathophysiologic entity is distinct from resting MR and can only be accurately quantified during exercise [2]. Notably, exercise-induced MR is associated with a greater risk of worsening heart failure and cardiovascular death [3,4].

**Figure 1:**
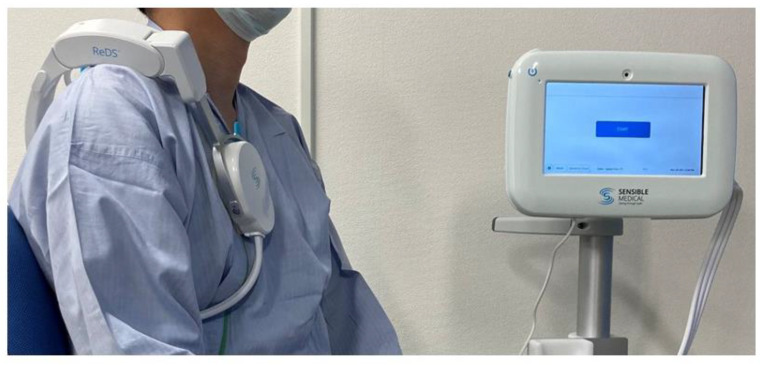
ReDS system. ReDS, remote dielectric sensing.

Exercise-induced MR is best assessed by stress echocardiography [1], during which the degree of pulmonary congestion might vary independently of the degree of resting MR. The Remote Dielectric Sensing (ReDS^TM^, Sensible Medical Innovations Ltd., Netanya, Israel) system, which is a novel electromagnetic-based methodology to quantify lung fluid volumes noninvasively [5], has recently been introduced (Figure 1). Prior investigations demonstrated a strong correlation between lung fluid levels and intra-cardiac filling pressures [6]. Furthermore, ReDS can quantify lung fluid volumes with comparable accuracy to high-resolution computerized chest tomography [7].

Change in ReDS values during stress echocardiography might provide additional information to consider potential valve repair in those with suspected exercise-induced MR in addition to conventional MR quantification with effective regurgitant orifice area (EROA).

## 2. Case Presentation

### 2.1. Participant Selection

Patients who were suspected of worsening exercise-induced MR underwent bicycle ergometer-utilized stress echocardiography testing between September 2021 and January 2022. Those with dyspnea due to other etiologies were excluded by the screening processes using computed tomography, spirometry, and laboratory data. Patients included were being considered for percutaneous valve repair given their frailty and anatomical feature of the mitral valves. ReDS values were measured at baseline and following the conclusion of the exercise protocol as detailed below. The institutional ethical review board approved the protocol and all participants signed informed consent.

### 2.2. Exercise Echocardiography

After two-dimensional and Doppler echocardiography was performed at rest, all patients performed symptom-limited bicycle ergometer exercises in the semi-supine position on a dedicated tilting table. The exercise was initiated at 10 Watts and the workload was maintained for 3 min. Thereafter, the workload was increased by 10 Watts every 3 min. Echocardiographic data including EROA were recorded continuously during the exercise. A change in EROA ≥0.10 cm^2^ was considered to be significant.

### 2.3. ReDS System

ReDS values were measured at rest and after the exercise testing protocol. The ReDS system has been described in-depth previously [7]. ReDS employs low-power electromagnetic signals emitted between two sensors embedded in wearable devices (Figure 1). ReDS estimates the lung fluid volume as a percentage.

### 2.4. Upon Presentation

A total of four patients who were suspected of exercise-induced MR were included (patients A–D in Table 1). All patients were above 70 years old and 3 were women. Left atrial diameter was above 50 mm in all patients. All patients had preserved left ventricular function. Their baseline MR was graded as mild to moderate. Tricuspid regurgitation was at least mild in all patients. Plasma B-type natriuretic peptide levels ranged between 79 pg/mL and 291 pg/mL.

### 2.5. Stress Echocardiography

**Figure 2:**
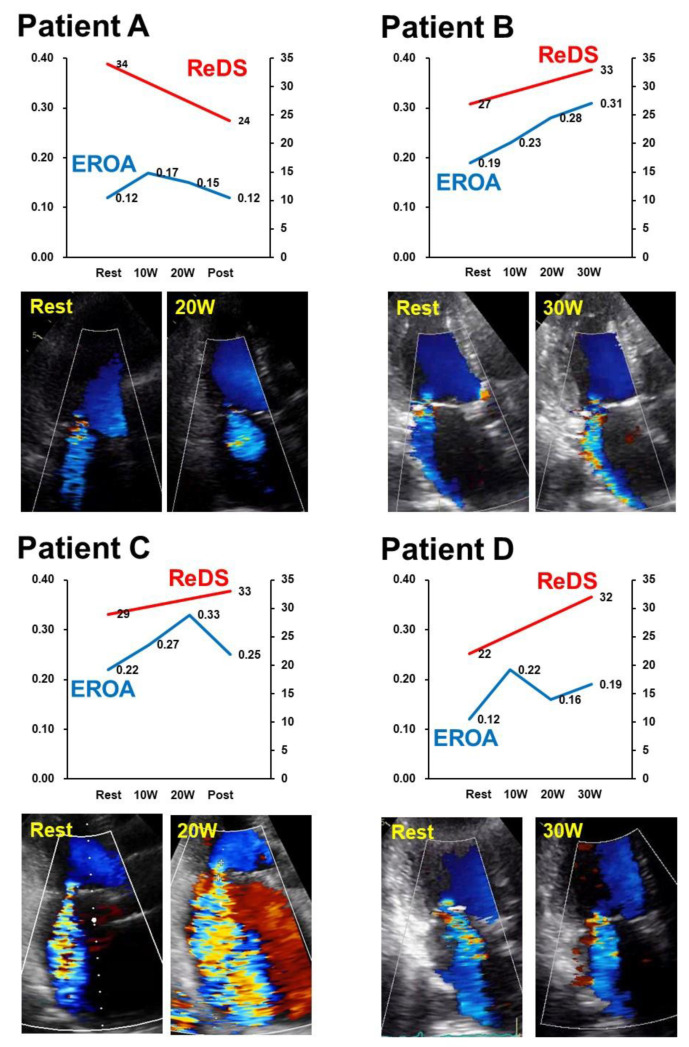
Trends of ReDS values and EROA during stress testing in the included four cases (**A**–**D**). ReDS, remote dielectric sensing; EROA, effective regurgitant orifice area.

At baseline, EROA ranged between 0.12 cm^2^ and 0.22 cm^2^ in all four participants (Figure 2A–D). All patients completed stress echocardiography using a bicycle ergometer without any complications.

Patient A completed bicycle ergometer up to 20 Watts. EROA remained around 0.15 cm^2^. ReDS value rather decreased from 34% to 24% (Figure 2A).

Patient B completed bicycle ergometer up to 30 Watts. EROA increased from 0.19 cm^2^ to 0.31 cm^2^. ReDS value also slightly increased from 27% to 33% (Figure 2B).

Patient C completed bicycle ergometer until 35 Watts. EROA increased from 0.22 cm^2^ to 0.33 cm^2^. ReDS value also increased slightly from 29% to 33% (Figure 2C).

In patient D, EROA increased slightly from 0.12 cm^2^ to 0.19 cm^2^ during the stress test (Figure 2D). ReDS value increased considerably from 22% to 32%.

### 2.6. Post-Test Course

All patients underwent percutaneous edge-to-edge mitral valve repair (MitraClip) except for patient A, who was continued on medical therapy. In patient D, the increase in EROA was not considerable. However, given daily severe dyspnea upon exertion and a low 6 min walk distance (<300 m) along with a considerable increase in ReDS value during exercise, the patient was referred for MitraClip.

**Figure 3:**
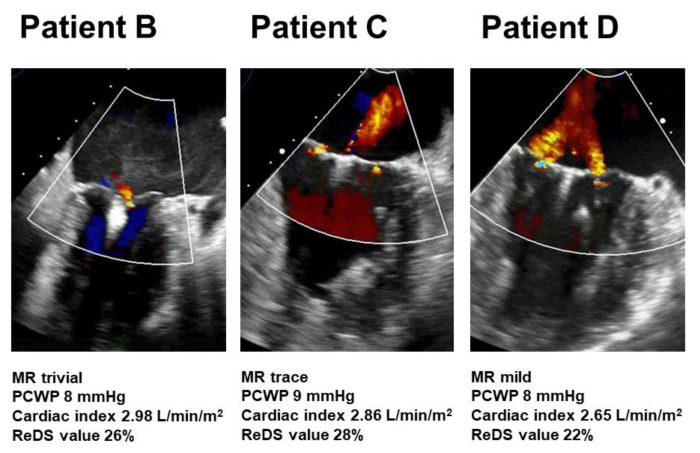
Transesophageal echocardiography following MitraClip procedure in patients B–D. MR, mitral regurgitation; PCWP, pulmonary capillary wedge pressure; ReDS, remote dielectric sensing.

Patient A was medically managed without any complications. Other patients received MitraClip procedure 39 days, 12 days, and 8 days following testing without any complications. Transesophageal echocardiography images in patients B–D following MitraClip procedures are displayed in Figure 3. The degree of residual MR ranged between trivial and mild. Their hemodynamics data and ReDS values were within acceptable ranges. Of note, patient D showed mild residual MR but the ReDS value was only 22%.

## 3. Discussion

Stress echocardiography can distinguish a specific high-risk cohort in those with less than severe MR at rest through evaluation of MR severity during exercise [2]. Of note, stress echocardiography is indicated for those with severe dyspnea upon exertion, despite mild to moderate baseline MR, to consider potential corrective interventions with significant increases in MR with exercise [1].

However, we also encounter patients who complain of severe dyspnea despite no change or only mild increases in MR EROA during exercise tests. We hypothesized that EROA may not completely inform us of the hemodynamic impacts of MR and, thus, a metric of quantifying pulmonary congestion may offer further prognostic information [8]. Echocardiographic presence of peak tricuspid valve regurgitant gradient may be one additional option to estimate pulmonary hypertension [9]. However, echocardiography cannot assess lung fluid volumes directly. This is a rationale to consider using the ReDS system to non-invasively quantify lung fluid levels during exercise.

It is not surprising that ReDS values increased mildly in patient B and patient C in addition to incremental changes in EROA during exercise. Worsening MR may lead to increases in left atrial pressure and progression of pulmonary congestion. On the contrary, ReDS value increased considerably in patient D despite the only mild increase in EROA during exercise. The detailed mechanism in this case remains uncertain, but the residual left atrial “reservoir” to compensate for incremental changes in left atrial pressure as well as impaired pulmonary vasculature elastance and permeability might be associated with an inappropriate increase in lung fluid volume, despite the slight change in EROA during exercise.

Percutaneous mitral valve repair using the MitraClip system conferred a greater event-free survival compared to medical therapy in patients with exercise-induced MR [10]. We proceeded with MitraClip for patient D despite a mild increase in EROA given a considerable increase in ReDS values during exercise. The implications of concomitant measurement of ReDS values during stress echocardiography tests in determining the indication of mitral valve intervention require further larger-scale studies.

Lastly, we measured ReDS values also following MitraClip procedures. Needless to say, the accurate quantification of MR is challenging following MitraClip procedures due to their atypical double regurgitation. Although further studies are warranted, ReDS technology might also help assess the impact of residual MR following MitraClip on pulmonary congestion. For example, although patient D had visually mild MR following the MitraClip procedure, its impact on pulmonary congestion, which was assessed by ReDS, was trivial.

### Limitations

We present only four patients as preliminary proof-of-concept data without any statistical analyses. No studies, including the present one, have assessed the association between hemodynamics data and ReDS values during exercise. Our observation and hypothesis should be validated in future larger-scale studies.

## 4. Conclusions

The ReDS system might be an additive tool to consider the indication of mitral valve intervention in addition to conventional stress echocardiographic assessment in patients with suspected exercise-induced MR, although larger-scale studies are warranted to validate our hypothesis.

## Figures and Tables

**Table 1:** Baseline characteristics.

	A	B	C	D
Demographics				
Age, years	83	74	83	84
Sex	Woman	Man	Woman	Woman
Body mass index	24.8	20.3	24.6	17.9
Ischemic etiology	None	None	None	None
Echocardiography				
Left atrial diameter, mm	53	53	64	56
Left ventricular end-diastolic diameter, mm	44	46	44	51
Left ventricular ejection fraction, %	73	55	61	55
Mitral regurgitation, grade	Mild to Moderate	Mild to Moderate	Mild to Moderate	Mild to Moderate
Tricuspid regurgitation, grade	Moderate	Mild	Moderate	Mild
Laboratory data				
Serum albumin, g/dL	4.0	3.5	3.0	4.6
Estimated glomerular filtration ratio, mL/min/1.73m^2^	26.9	64.9	43.2	56.7
Plasma B-type natriuretic peptide, pg/mL	79	291	256	252
Medication				
Beta-blocker	Yes	Yes	Yes	Yes
Renin-angiotensin system inhibitor	Yes	Yes	Yes	Yes
Mineralocorticoid receptor antagonist	No	Yes	No	No
Diuretics	Yes	Yes	Yes	No
Inotropes	No	No	No	No

## Data Availability

Data are available upon appropriate reasons.
